# The Predictive Value of the Cholesterol-to-Natural Killer Cell Ratio in Colorectal Cancer

**DOI:** 10.7150/jca.114813

**Published:** 2025-06-23

**Authors:** Qian-Qian Liu, Yan Chen, Zhi-Qing Zhan, Hao-Lian Wang, Ying-Xuan Chen

**Affiliations:** 1 Division of Gastroenterology and Hepatology, Key Laboratory of Gastroenterology and Hepatology, Ministry of Health, State Key Laboratory for Oncogenes and Related Genes, Shanghai Institute of Digestive Disease, Renji Hospital, School of Medicine, Shanghai Jiao Tong University, Shanghai, China.; 2 Department of Gastroenterology, Sichuan Provincial People's Hospital, School of Medicine, University of Electronic Science and Technology of China, Chengdu, China.

**Keywords:** Colorectal cancer, Cholesterol, NK cell, Tumor markers

## Abstract

**Purpose:** We constructed a novel biomarker cholesterol (C)-to-natural killer (NK) cell ratio (CNR) to reflect the synergistic effect of cholesterol metabolism and inflammation on colorectal cancer (CRC) outcomes. This study aimed to investigate the clinical significance and predictive value of CNR in CRC and develop a simple and reliable prognostic model for predicting OS in CRC patients.

**Methods:** We retrospectively collected the hematology data and medical records of 213 patients with CRC at Renji hospital and the histological data and medical records of 94 patients with CRC included in a tissue microarray. The association between tumor biomarkers and survival was evaluated using the log-rank test. The diagnostic efficacy of CNR was assessed using receiver operating characteristic curves. The overall survival (OS) rates were estimated using the Kaplan-Meier method. Cox proportional hazards regression was employed in both univariate and multivariate analyses to identify independent prognostic factors, which were subsequently utilized to develop a predictive model for OS. The performance of the model was evaluated using the concordance index (C-index) and calibration plots. The patients were stratified based on the total risk scores calculated from the model. The differences in OS among these groups were assessed using the Kaplan-Meier method. The relationship between cholesterol and NK cells was analyzed by investigating the colon cancer datasets TCGA and GSE39582.

**Results:** The TNM stage III-IV CRC group had significantly higher blood levels of cholesterol, triglycerides, low-density lipoprotein cholesterol (LDL-C), CNR, and carcinoembryonic antigen (CEA), and shorter progression-free survival (PFS) than the TNM stage I-II CRC group (all, *P* < 0.05). The blood CNR correlated negatively with PFS (*P* < 0.001). Elevated tissue CNR levels were an independent risk factor for CRC, where low-tissue CNR patients demonstrated significantly prolonged survival (*P* < 0.05). The area under the curve for blood CNR was 0.743. The multivariate analyses indicated that tumor location (*P* = 0.004), TNM stage (*P* = 0.004), and tissue CNR (*P* = 0.033) were independent prognostic factors of OS in CRC. The nomogram model was based on these variables and demonstrated good calibration and predictive performance, achieving an excellent C-index of 0.737 [95% confidence interval (CI), 0.674-0.779]. The expression of the key cholesterol biosynthesis players *HMGCR*, *SQLE*, and *SREBP2* was not significantly associated with NK cell-mediated cytotoxicity-related gene signatures. *HMGCR* and *SQLE* were negatively associated with *CD56*, a phenotypic NK cell marker (*P* < 0.001).

**Conclusion:** This is the first study to explore the predictive value of CNR in CRC, which was a promising predictor of CRC progression. The developed nomogram model may serve as a reliable tool for predicting survival in patients with CRC, which may complement the TNM staging system.

## Introduction

Colorectal cancer (CRC) is one of the most prevalent malignant tumors worldwide and a leading cause of cancer-related deaths 1, 2. GLOBOCAN data stated that there were more than 1.9 million new cases of CRC and more than 900,000 deaths worldwide in 2020. While developed countries have a higher CRC incidence, its incidence in some developing nations is also increasing 3, 4. Age, gender, genetic predispositions, and lifestyle choices, including high-fat and high-red meat diets, obesity, smoking, and lack of exercise, significantly influence CRC risk. Despite advancements in surgical treatment and chemoradiotherapy, the long-term outcomes for patients with CRC remain concerning. Various factors affect CRC prognosis, including tumor staging, degree of differentiation, lymph node metastasis, and biomarker status, such as microsatellite instability 5-8. However, the predictive performance of these factors remains unsatisfactory due to the high heterogeneity of CRC. Consequently, novel prognostic indicators and models are essential for improving the identification of patients at higher risk of mortality, allowing for more tailored treatment planning.

A notable characteristic of cancer cells is their metabolic abnormalities, including lipid metabolism alterations 9,10. Recent studies have identified a complex relationship between serum lipids, such as total cholesterol, high-density lipoprotein cholesterol (HDL-C), triglycerides, and cancer occurrence and progression 11-14. CRC cells have elevated cholesterol uptake and accumulation, promoting their proliferation. Furthermore, cancer occurrence and progression appear to be linked to the consumption of circulating cholesterol by the tumor 15-17. Additionally, systemic inflammatory responses are crucial in cancer development, as cancer-associated inflammatory mediators contribute to immune suppression and immune escape 18-20. Immune and inflammation-related biomarkers, such as lymphocyte counts, neutrophil-to-lymphocyte ratio (NLR), lymphocyte-to-monocyte ratio (LMR), and platelet-to-lymphocyte ratio (PLR), are commonly used to predict CRC long-term prognosis 21-27. Recent studies have also demonstrated that the accumulation of cholesterol and its metabolites in the tumor microenvironment (TME) positively affect immune suppression. For example, inflammation-induced lipid-laden lung mesenchymal cells metabolically reprogram tumor cells and natural killer (NK) cells, facilitating breast cancer lung metastasis 28. However, a study that examined the prognostic value of cholesterol and its relationship with immune signatures did not assess the combined effect of cholesterol metabolism and NK cells on the prognosis of patients with CRC 29. Therefore, this study aimed to evaluate the prognostic value of the cholesterol-to-NK cell ratio (CNR) in CRC and develop and validate a novel prognostic model that may complement the TNM staging system.

## Materials and methods

### Patient population

This study included patients diagnosed with CRC. Inclusion criteria for the CRC group: (1) CRC confirmed by histopathology; (2) patients did not receive surgery or any treatment. Exclusion criteria: (1) after undergoing surgery or treatment; (2) history of other tumors or malignancies; (3) serious infection and chronic disease; (4) incomplete clinical information, laboratory data, and follow-up data. The ethics committee of Renji Hospital, Shanghai Jiao Tong University School of Medicine (Shanghai, China) approved the data analysis (Approval no. KY2023-101-C). In accordance with institutional guidelines, informed consent was obtained from all patients before analysis.

### Data collection

Each patient's clinical stage (stage I-IV) was determined based on the 8th edition of the TNM Classification of Malignant Tumors published by the American Joint Committee on Cancer. The relevant clinical and histopathological characteristics provided to the researchers were anonymized. Detailed information, including demographic characteristics (age, gender, body mass index [BMI]), clinicpathological characteristics (cholesterol level, lymphocyte ratios, serum CEA), tumor-related characteristics, treatment, and oncological outcomes, was collected from each patient. All analyses were conducted in accordance with the provisions of the Helsinki Declaration of 1975.

### Immunohistochemistry

The tissues underwent immunohistochemical analysis to examine the NK cells. CD56 expression was detected in tissue microarrays (Outdo Biotech, Shanghai, China, HCo1A180Su22) containing 94 patients with colorectal cancer. Endogenous peroxidase activity was quenched with 3% H_2_O_2_ for 10 minutes, then the slide was washed and heated in a microwave with citrate buffer for antigen retrieval. Subsequently, the slide was blocked with horse serum for 30 minutes and incubated overnight at 4°C with primary antibodies against CD56 (Servicebio, Wuhan, China, GB112671, 1:500), followed by incubation with horseradish peroxidase-conjugated polyclonal anti-rabbit antibodies at room temperature for 30 minutes. Finally, the slide was developed using DAB buffer according to standard protocols. The immunohistochemical staining results were evaluated in a blinded manner (The evaluators were blinded to the clinical characteristics of individual patients). CD56 protein expression levels were quantified depending on staining intensity and extent (percentage of positive tumor cells) under a microscope (200×). Staining intensity score was evaluated on a scale of 0-3: 0 = no staining, 1 = weak staining, 2 = moderate staining, and 3 = strong staining. Further, staining extent score indicated the percentage of positively stained cells (0 = 0%-5%, 1 = 6%-25%, 2 = 26%-50%, 3 = 51%-75%, and 4 = 76%-100%). To derive the final score, staining intensity and extent scores were multiplied.

### Immunofluorescence

The tissue microarrays underwent immunofluorescence analysis to examine the total cholesterol (Haling Biotech, Shanghai, China, HL80079.2.1). Briefly, the slide was dewaxed, purified, cleaned, hydrolyzed, and sealed, then stained for 30 minutes, where the fluorescent signal for cholesterol appeared blue. The fluorescence was analyzed using a panoramic scanning microscope (3D HISTECH, Hungary), and digital image processing was performed using CaseViewer 2.4.

### Statistical analysis

Count data are expressed as the frequency and percentage, while measured data are expressed as means. Data were compared between groups using the independent samples t-test or the Mann-Whitney U test. Correlations were analyzed using Spearman correlation analysis. The correlation between clinical features and PFS or OS was investigated using the Kaplan-Meier method and log-rank test. Potential prognostic factors were identified using univariate and multivariate analysis with Cox proportional hazards regression. Significant factors identified in the multivariate analysis were used to construct a nomogram model predicting the patients' 3- and 5-year OS rates. Gene set enrichment analysis (GSEA) was assessed using sangerbox (http://sangerbox.com/home.html). *P* < 0.05 was considered statistically significant.

## Results

### Pathological characteristics of CRC patients

This study initially enrolled 213 patients with CRC, of which 27 (12.7%) were TNM stage I-II and 186 (87.3%) were stage III-IV. The correlation between TNM stage and clinicopathological factors (gender, age, BMI, tumor size, tumor location, histology, blood lipids) was evaluated (Table 1). Patients in the TNM stage III-IV group had significantly larger tumor size and higher serum CEA, cholesterol, triglyceride, and LDL-C levels than those in the TNM stage I-II group (*P* = 0.029, 0.040, 0.017, 0.044, 0.032, respectively), while the TNM stage III-IV group had shorter PFS than the stage I-II group (*P* = 0.023).

### The association between clinical factors and PFS

**Figure 1:**
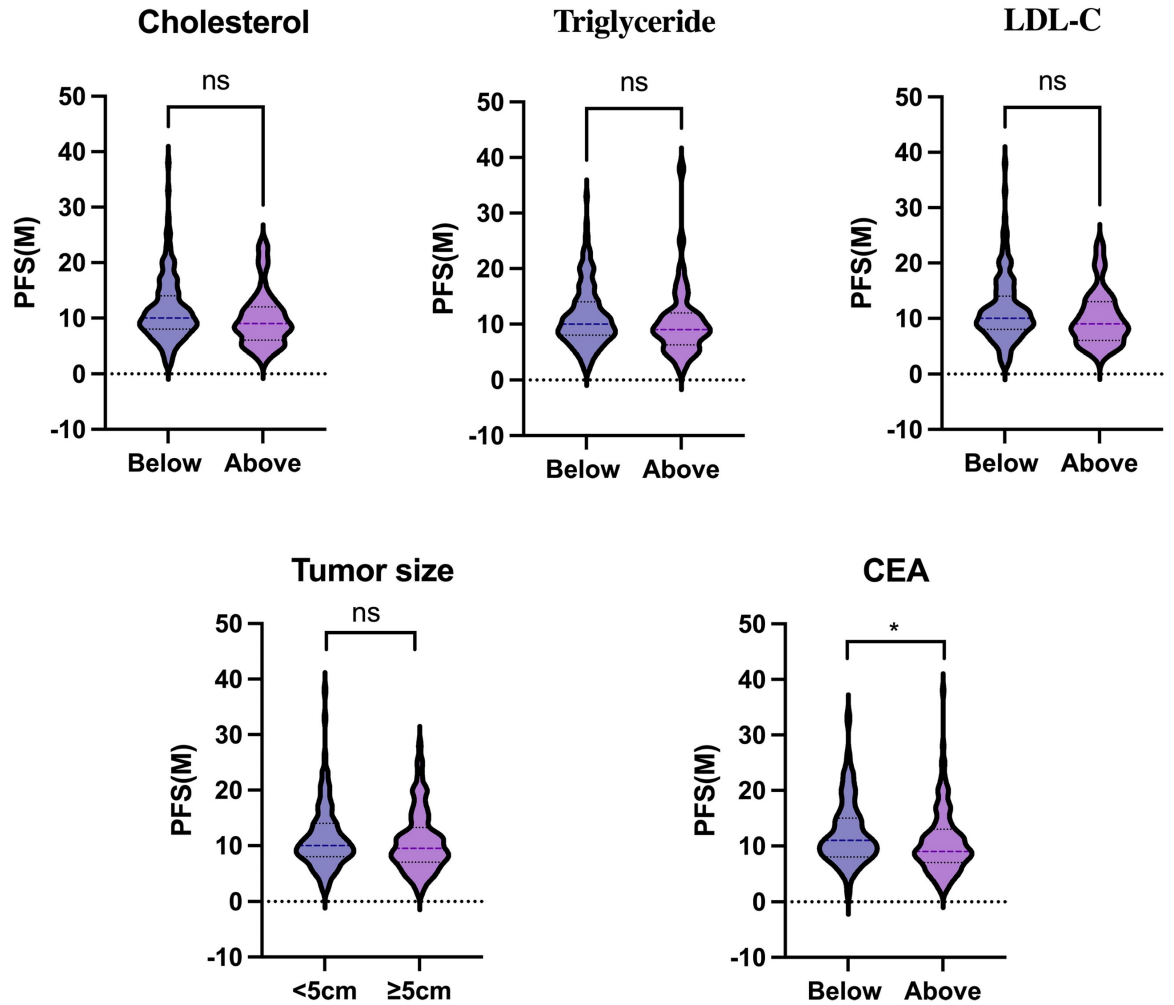
The association between clinical factors (blood lipids, tumor size, serum CEA) and PFS. Statistical significance was determined by unpaired Student's t test. *, *P* < .05.

We further investigated the association between the above clinical factors and PFS. Patients were divided into two groups based on their blood cholesterol, triglyceride, LDL-C or CEA levels (below or above the upper limit) or tumor size (< or ≥5cm). Our results indicated that blood cholesterol (*P* = 0.075), triglyceride (*P* = 0.318), LDL-C (*P* = 0.074), and tumor size (*P* = 0.426) were not significantly associated with PFS (Fig. 1). However, patients with low serum CEA levels had a longer PFS compared to those with high CEA levels (*P* = 0.035).

### Inflammatory biomarker analysis

**Figure 2:**
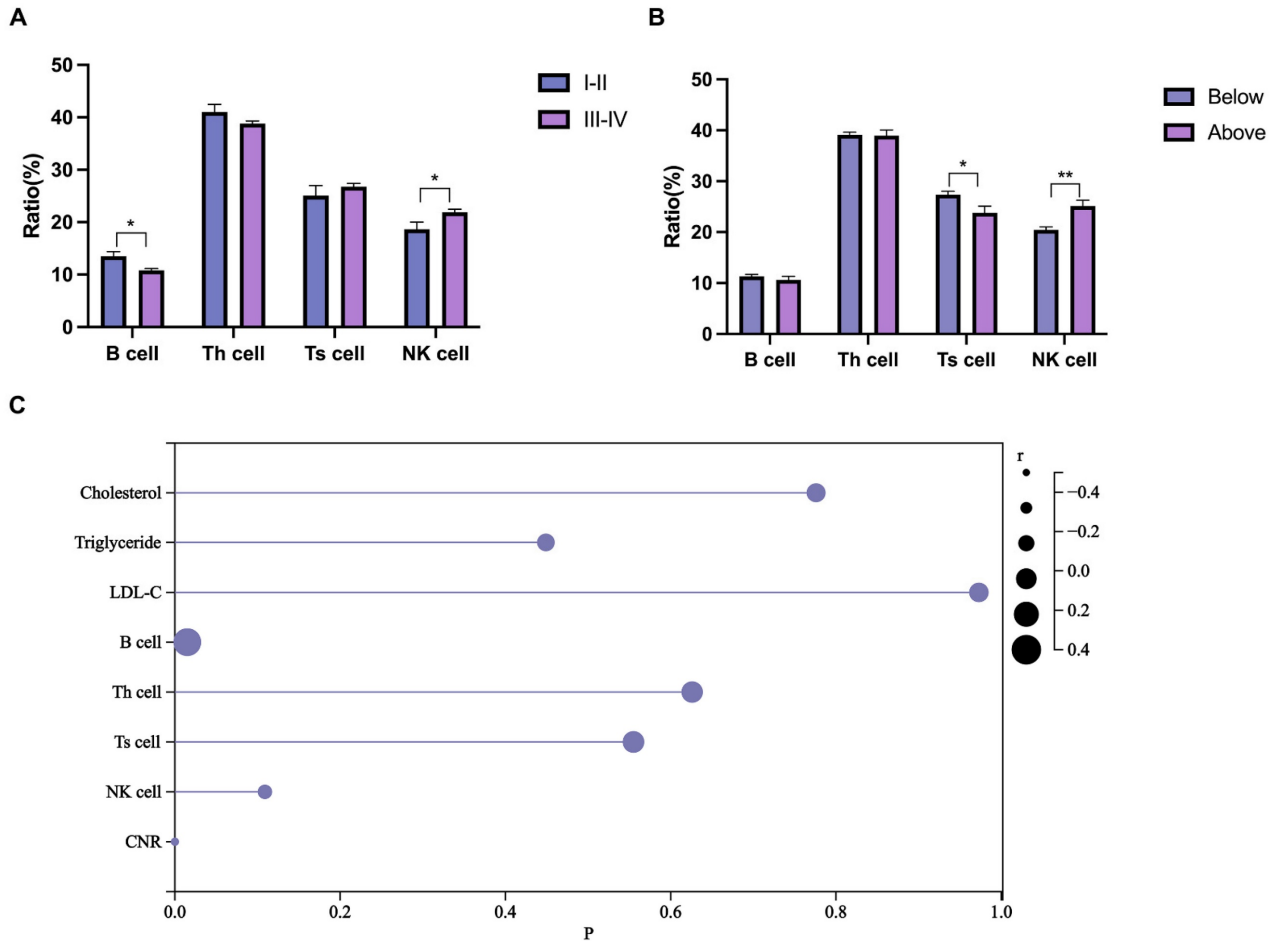
Inflammatory biomarker analysis. **(A)** Differences in blood lymphocyte ratios between patients with stage I-II CRC and patients with stage III-IV CRC. **(B)** Differences in blood lymphocyte ratios between high- and low-cholesterol (blood) patients with CRC. **(C)** Lollipop chart of the correlation of blood lipids, lymphocytes, and CNR with PFS. Data are expressed as the mean ± SEM (A and B). Statistical significance was determined by unpaired Student's t test (A and B) and Spearman's rank-sum test (C). *, *P* < .05; **, *P* < .01.

The systemic inflammatory response is vital in cancer. Compared to TNM stages I-II, the TNM stage III-IV group had a reduced B cell ratio and increased NK cell ratio, while the T helper (Th) and T suppressor (Ts) cell ratios remained unchanged (Fig. 2A). To further investigate the synergistic effect of blood lipids on the lymphatic system, patients with colorectal cancer were divided into two groups based on their blood cholesterol, triglyceride, or LDL-C levels (below or above the upper limit). Patients in the high-cholesterol group (above the upper limit) had a higher NK cell ratio and a lower Ts cell ratio (Fig. 2B), while no effect was demonstrated for triglycerides (Supplementary Fig. S1A) and LDL-C levels (Supplementary Fig. S1B). Combined effect of cholesterol metabolism and lymphocyte on the prognosis is promising 29. We then analyzed the correlation of blood lipids and lymphocyte ratios with PFS of CRC patients. The Spearman correlation analysis (Fig. 2C) demonstrated significant associations between B cells (r = 0.324, *P* = 0.015) and the CNR (r = -0.468, *P* < 0.001) with PFS.

### The correlation between blood CNR and clinical factors

The blood CNR was classified into low and high levels based on the optimal cut-off point of 29.99, as determined by BioLadder software (Supplementary Fig. S2), which yielded an area under the curve of 0.743. The correlation between the blood CNR and clinicopathological factors (gender, age, BMI, tumor size, tumor location, histology) was evaluated (Table 2). Notably, the blood CNR-high group had a poorer pathological stage than the blood CNR-low group (*P* = 0.041). Furthermore, the blood CNR-high group had a significantly shorter PFS than the blood CNR-low group (*P* = 0.003). The results indicated that blood CNR may be a promising predictor in CRC. Additionally, the proportions of females in the blood CNR-high group were significantly higher than those in the blood CNR-low group (*P* = 0.021). Why blood CNR is influenced by gender remains unclear. A large-scale study and additional analyses are needed to confirm these findings.

### The correlation of tissue CNR with OS in human colorectal cancer

**Figure 3:**
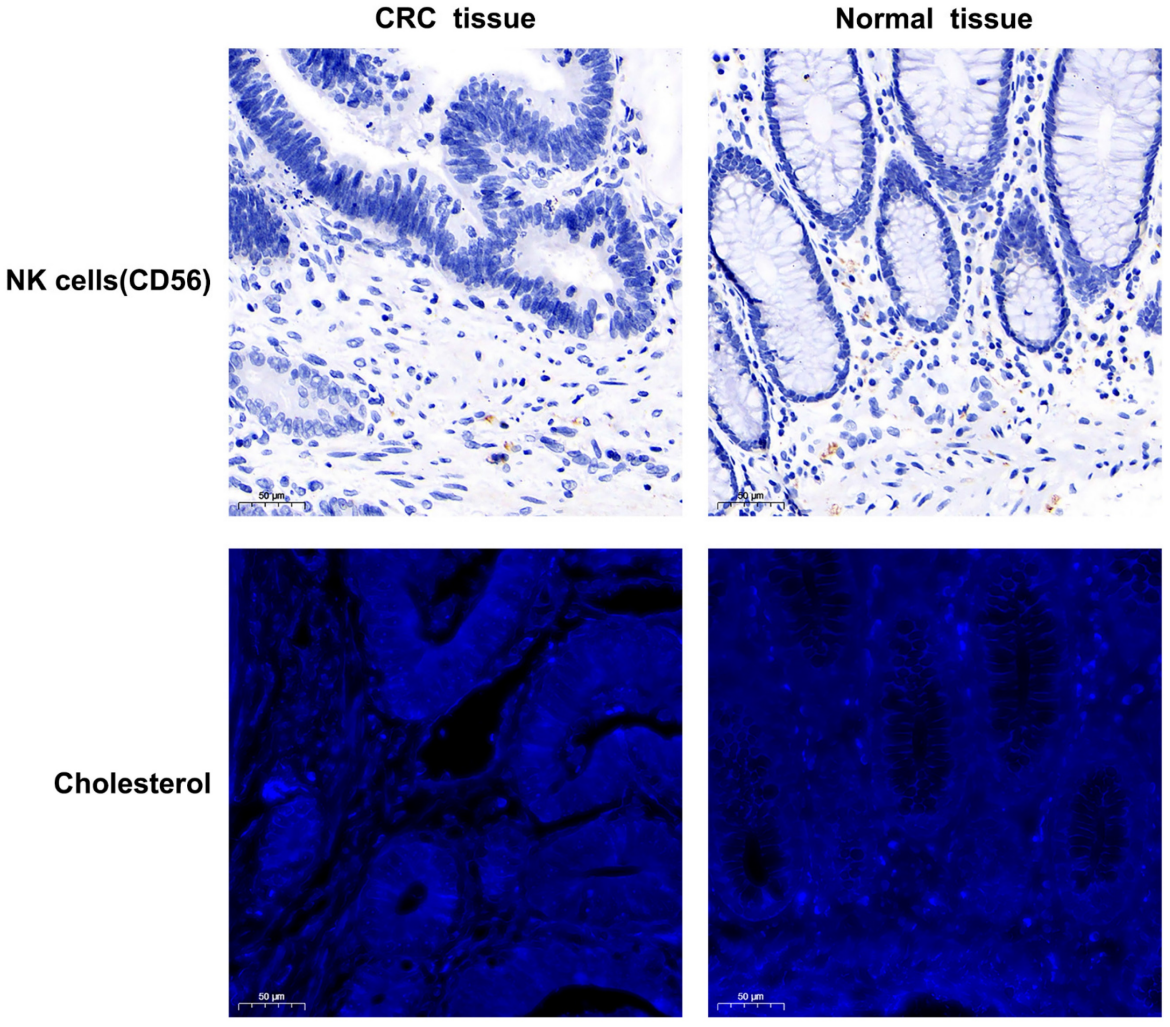
The NK cells (tissue, immunohistochemical staining) and cholesterol (tissue, immunofluorescence staining) expression profiles in human colorectal cancer samples (200× magnifications).

**Figure 4:**
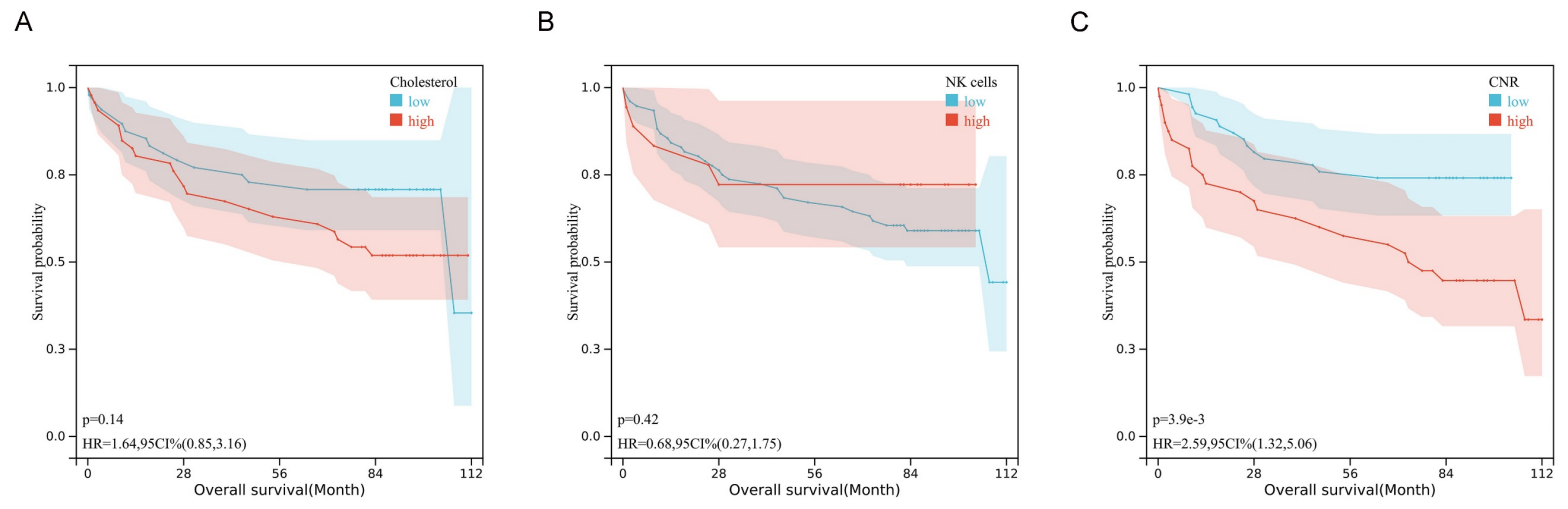
Kaplan-Mier survival curves of the OS in patients with CRC stratified by tissue cholesterol **(A)**, tissue NK cells **(B)**, and tissue CNR **(C)**.

The tissue expression profiles of cholesterol and NK cells in human colorectal cancer were investigated through immunofluorescence staining and immunohistochemical staining in 94 colorectal cancer specimens. TNM stage I, II, III, and IV disease was observed in 7 (7.4%), 44 (46.8%), 40 (42.6%), and 3 (3.2%) of the patients, respectively. The clinicopathological data are presented in Supplementary Table 1 and Supplementary Table 2. Notably, the tissue CNR-high group had a significantly poorer survival rate and shorter OS than the tissue CNR-low group (*P* = 0.003, 0.031, respectively). High tissue CD56 expression (indicative of NK cells) or cholesterol was observed in 18 (19.1%) and 46 (48.9%) of the 94 specimens, respectively. Figure 3 depicts representative images of the tissue CD56 immunohistochemical staining and the tissue cholesterol immunofluorescence staining. The human CRC tissue had lower NK cell and cholesterol levels than the normal tissue. However, the data showed no significant differences in the expression of tissue NK cells and cholesterol across TNM stages I to IV, which might have been limited by the relatively small sample size (Supplementary Fig. S3). The Kaplan-Meier curves revealed that tissue cholesterol (*P* = 0.14) and NK cells (*P* = 0.42) expression was not significantly associated with OS (Fig. 4). However, patients with low tissue CNR had a longer OS compared to those with high tissue CNR (*P <* 0.001).

### Prognostic factors in the univariate and multivariate analyses

The effect of clinicopathological characteristics on survival was investigated using Cox proportional hazards regression (Table 3). The univariate analysis results revealed that tumor location (left vs. right, *P* = 0.021), TNM stage (III-IV vs. I-II, *P* = 0.011), and tissue CNR (high vs. low, *P* = 0.008) were significantly associated with OS. Furthermore, the multivariate analysis confirmed that tumor location (left vs. right, hazard ratio [HR]: 2.811, 95% CI [1.414-5.794], *P* = 0.004), TNM stage (III-IV vs. I-II, HR: 2.722, 95% CI [1.382-5.543], *P* = 0.004), and tissue CNR (high vs. low, HR: 2.493, 95% CI [1.106-5.997], *P* = 0.033) were significant independent predictors of OS. The predictive ability of the tissue CNR and TNM stage was compared using the concordance index (C-index). The C-index of tissue CNR and TNM stage was 0.661 and 0.635 respectively, with no statistically significant difference. Wang's team compared the prognostic value of TNM stage and NLR in patients with colorectal cancer 29. Results showed that the C-index of TNM stage and NLR was 0.663 and 0.551 respectively, which corroborates the superior predictive value of CNR.

### Prognostic nomogram model

**Figure 5:**
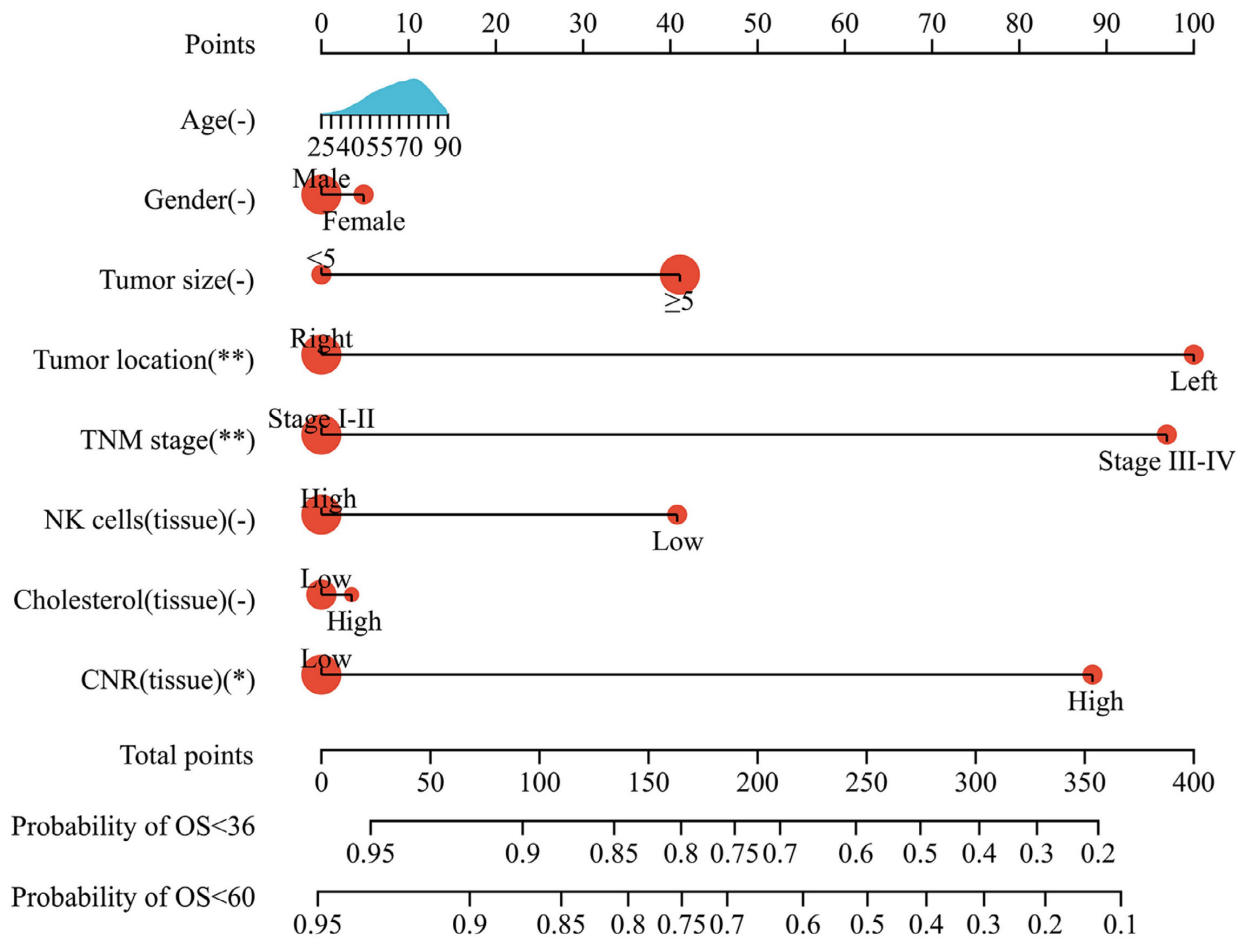
Nomogram model for predicting the OS of patients with CRC.

Based on the multivariate analysis results, a nomogram model incorporating tumor size, tumor location, TNM stage, and tissue CNR was constructed to predict OS in CRC patients (Fig. 5). Each variable was assigned a point. The patients' prognosis was assessed using a total risk score. The nomogram model was transformed from an online calculator, sangerbox 30, where the corresponding predicted survival rate was obtained by inputting clinical factors and selecting a time point.

The predictive ability of the nomogram and non-tissue CNR nomogram was compared using the C-index (Table 4). The C-index of the nomogram was 0.737, which indicated a good predictive accuracy. Furthermore, the comparison demonstrated that incorporating the tissue CNR significantly enhanced the predictive performance (C-index: nomogram: 0.737, 95%CI [0.674-0.779] vs. non-tissue CNR nomogram: 0.692, 95%CI [0.620-0.765], *P* < 0.05). Additionally, the calibration curves demonstrated that the predicted survival rates calculated by the nomogram model closely aligned with the actual observed values, suggesting strong consistency between the predictions and real-world outcomes (Supplementary Fig. S4).

**Figure 6:**
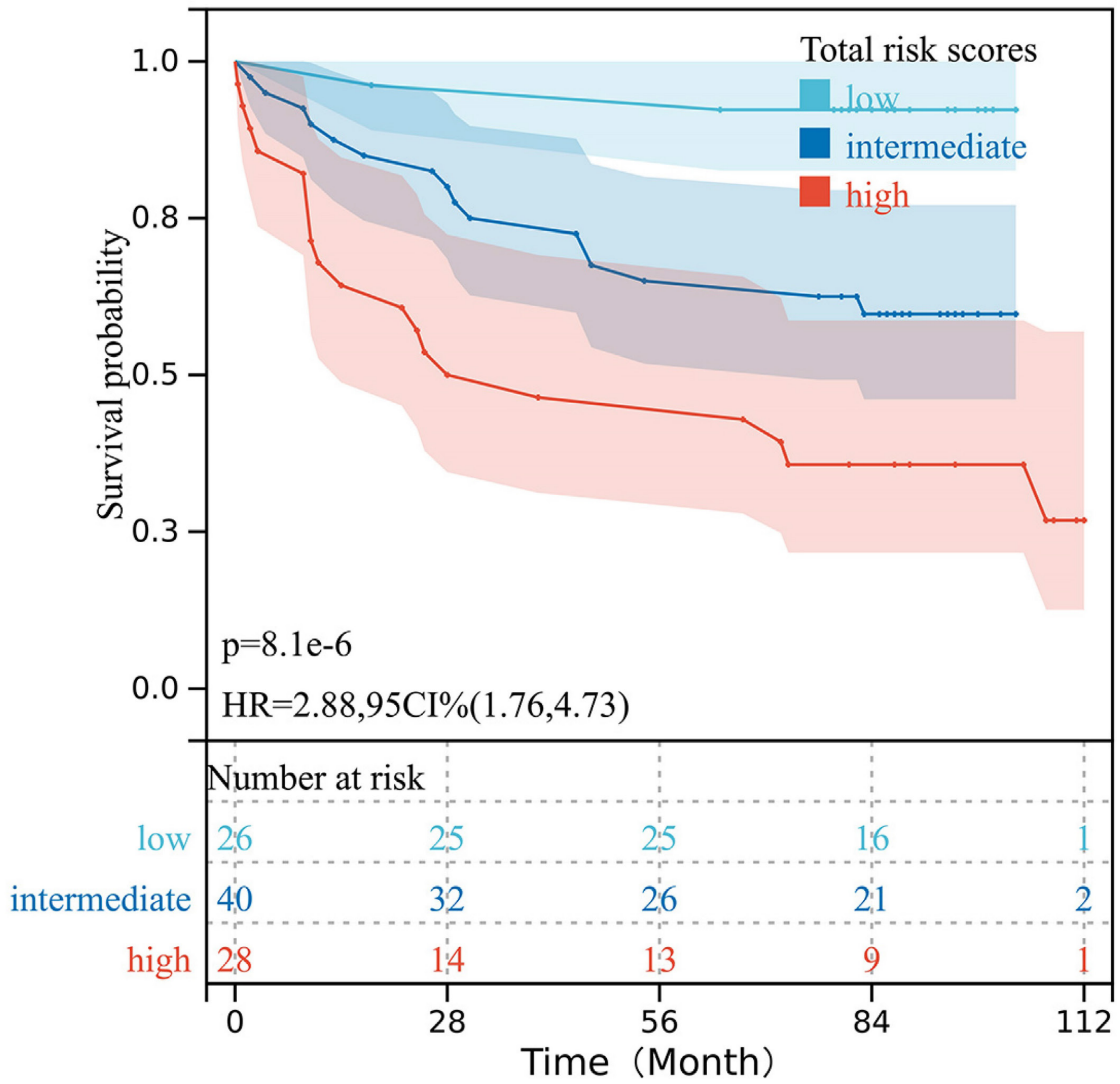
Survival curves for the OS of patients with CRC stratified according to the total risk score obtained from the nomogram model.

The total risk scores of all 94 patients with CRC were obtained based on the prognostic nomogram model. All risk scores were arranged in ascending order, then points were set at the 30% and 70% percentiles as the cut-off values, and the patients were categorized into low-, intermediate-, and high-risk groups. The Kaplan-Meier analysis indicated that patients with different risk levels had significantly different OS (*P* < 0.001) (Fig. 6).

### Gene expression of cholesterol synthesis and NK cells in colon cancer datasets

**Figure 7:**
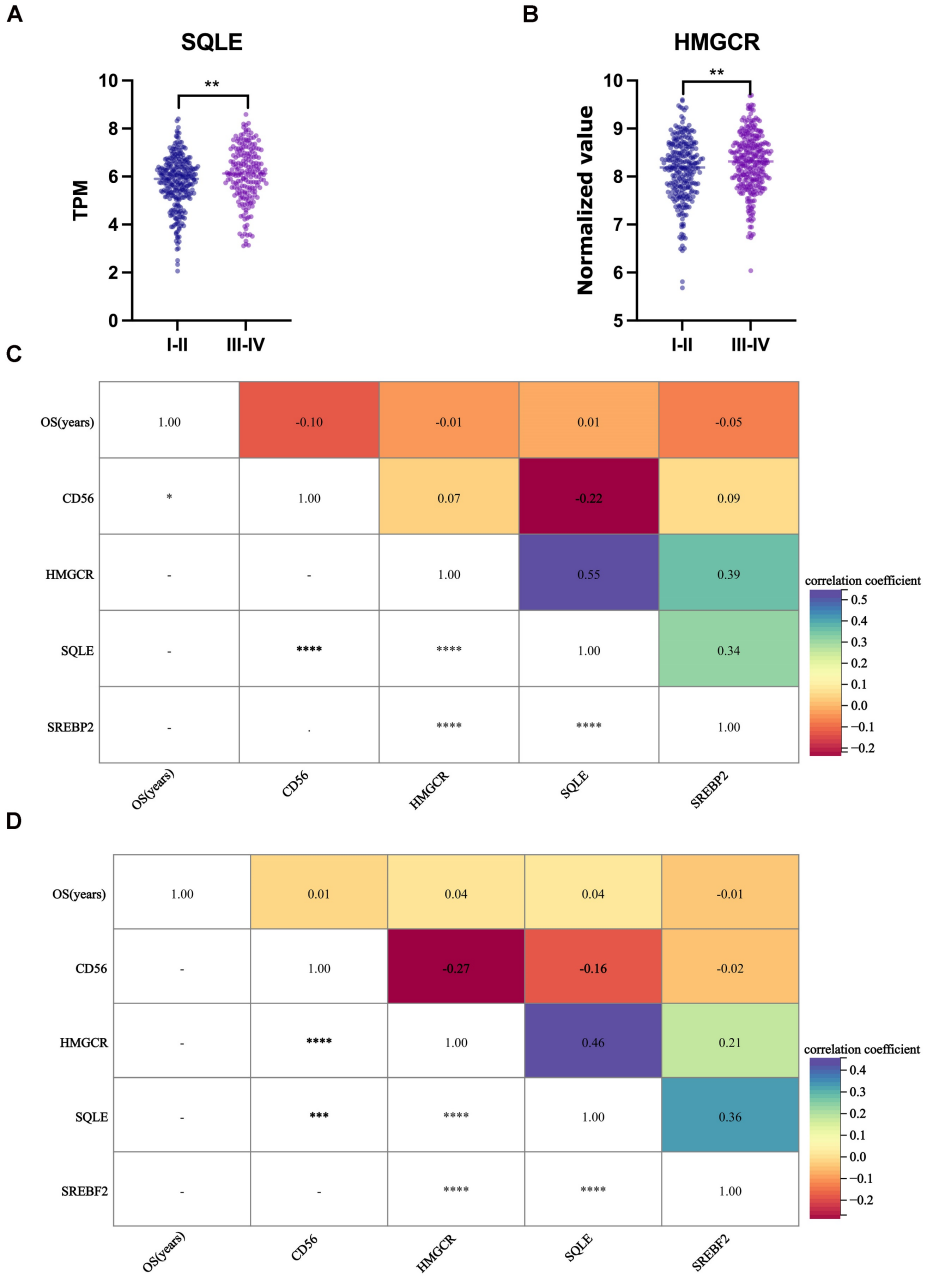
Gene expression of cholesterol synthesis and NK cells in colon cancer datasets.** (A)** *SQLE* gene expression was increased in the TNM stage III-IV group compared to the TNM stage I-II group (TCGA). **(B)** *HMGCR* gene expression was increased in the TNM stage III-IV group compared to the TNM stage I-II group (GSE39582). **(C)** *CD56* and *SQLE* were negatively related (TCGA). **(D)** *CD56* and *SQLE* or *HMGCR* were negatively related (GSE39582). Statistical significance was determined by unpaired Student's t test (A and B) and Spearman's rank-sum test (C and D). **, *P* < .01; ***, *P* < .001; ****, *P* < .0001.

The cellular cholesterol level reflects the dynamic balance between biosynthesis, uptake, export, and esterification 31. The relationship between cholesterol and NK cells was investigated by analyzing two independent colon cancer datasets (The Cancer Genome Atlas [TCGA], 475 colon cancer cases; GSE39582, 558 colon cancer cases). Almost all cells can synthesize cholesterol; therefore, we briefly examined three crucial players of the cholesterol biosynthetic pathway: two rate-limiting enzymes of the biosynthetic pathway: 3-hydroxy-3-methylglutaryl coenzyme A reductase (HMGCR) and squalene monooxygenase (SQLE), and sterol regulatory element-binding protein 2 (SREBP2), a master transcriptional regulator of cholesterol biosynthesis. Compared to the TNM stage I-II group, the TNM stage III-IV group had significantly increased gene expression of *SQLE* (Fig. 7A) and *HMGCR* (Fig. 7B) (*P* = 0.001, 0.005, respectively), while *CD56* and *SREBP2* gene expression was unchanged (Supplementary Fig. S5). Furthermore, *CD56* and *SQLE* (Figs. 7C, 7D) or *HMGCR* (Fig. 7D) were negatively related (r = -0.22, -0.27, respectively, *P <* 0.001).

**Figure 8:**
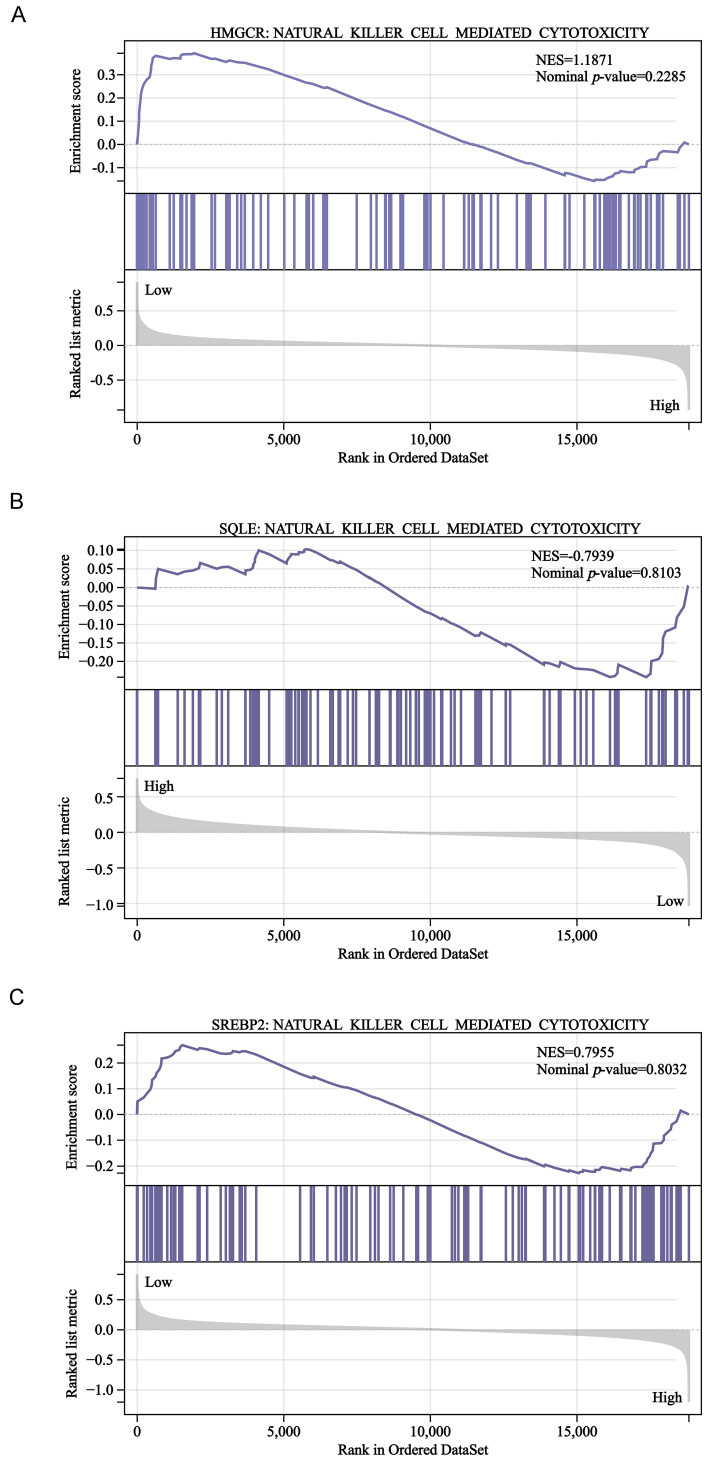
GSEA of the GSE39582 dataset. *HMGCR* **(A)**, *SQLE* **(B)**, and *SREBP2* **(C)** expression was not significantly associated with NK cell-mediated cytotoxicity- related gene signatures.

To further explore the association between cholesterol synthesis and NK cells, we performed GSEA in the GSE39582 dataset. However, our results revealed that *HMGCR*, *SQLE*, and *SREBP2* expression was not significantly associated with NK cell-mediated cytotoxicity-related gene signatures (Fig. 8A-C). Therefore, more studies are needed to confirm whether and how cholesterol regulates NK cells in CRC.

## Discussion

In the present study, more than 42% patients exhibited an elevated CNR, which we determined was significantly associated with decreased PFS or OS. To our knowledge, this is the first study to describe the correlation between CNR and survival rates in CRC. Therefore, pre-treatment CNR represents a novel biomarker that can effectively predict the prognosis of patients with CRC and can be a valuable supplement to the TNM staging system, aiding clinicians in more accurate treatment planning.

Cholesterol is an important cell membrane component and participates in signal transduction, cell proliferation, and apoptosis processes. Lipid disequilibrium, especially abnormal cholesterol metabolism, is closely related to various diseases, especially tumor occurrence and development 32,33. CRC is a common malignant tumor of the digestive system that is closely related to cholesterol metabolism 15,34-36. Reportedly, excessive intake of cholesterol is correlated with CRC occurrence. Mutations in the cholesterol metabolism-related genes are associated with an increased risk of CRC 37,38. While elevated cholesterol is associated with the incidence of colorectal adenomas and tumors, the conclusions on the relationship between serum cholesterol levels and CRC prognosis have been inconsistent. Ren *et al.* reported that statin use was associated with a significantly lower risk of incident cancer and cancer-related mortality 39. Conversely, another study reported that the statin-induced reduction of plasma cholesterol levels did not significantly affect recurrence and survival rates in patients with stage III cancer 40. In the present study, cholesterol alone had no predictive value for survival, but effectively distinguished CRC patients with poor prognosis when combined with immune markers.

The immune system has recently received extensive attention. Cholesterol and its metabolites within the TME are crucial in cancer progression, as they regulate cancer cell proliferation, migration, and invasion, and modulate immunity and inflammation 41-45. A high-cholesterol diet may promote inflammation and tumor formation by affecting the gut microbiota composition 46. Previous studies analyzed and combined several serum lipid indices and inflammation-related biomarkers to predict the prognosis of CRC. The cholesterol-to-lymphocyte ratio (CLR) and monocyte-to- HDL-C ratio (MHR) have been correlated with long-term prognosis in CRC 29,47. In the present study, the hematological data of 213 patients with CRC and the tissue microarray-based histological data of 94 patients with CRC revealed that patients at more advanced TNM stages had significantly higher CNR, which is dependent on the combination of cholesterol and NK cells. Additionally, the CNR level was identified as an independent prognostic factor of OS in CRC, and showed slightly superior prognostic value than TNM stage, which is one of the most widely used indictor for prognosis. All of the data indicated that CNR had a reliable prognostic value.

NK cells are integral innate immune system components, pivotal in anti-tumor immunity, and crucial in tumor immunology. Numerous studies have demonstrated that NK cells exert anti-tumor cytotoxic effects through specific functional receptors on their surface, such as NKG2 and the KIR family 48. NK cells secrete cytokines and chemokines, such as CCL5, XCL1, and XCL2, which promote dendritic cell migration into solid tumors, and enhance the anti-tumor activity of CD8^+^ T cells 49. Various substances within the TME influence the biological activity of NK cells. Lynch's team was the first to discover that obesity significantly induces the activation of peroxisome proliferator-activated receptors (PPARs) in NK cells, leading to fat accumulation and resulting in the failure of NK cell-mediated immune surveillance, thereby inhibiting their normal tumor cell-killing effects 50. Liu and colleagues confirmed that FASN, which was upregulated in CRC tissues, suppressed the antitumor response of NK cells through the SP1/PLA2G4B axis 51. Accordingly, it is important to consider the synergistic effects of cholesterol metabolism and NK cells on CRC prognosis. Our results demonstrated the predictive value of CNR and we have incorporated it into a prognostic model. This model, with a high accuracy and stability, is expected to provide a novel approach to optimizing risk stratification for patients with CRC.

Our study has several limitations. First, due to the modest sample size, we were unable to perform propensity score matching (PSM) to balance baseline characteristics, despite some differences observed between groups (e.g., gender, and tumor location). While PSM is a robust method for reducing selection bias, its application in small cohorts risks significant sample loss and compromised statistical power. Instead, we controlled for potential confounders through univariate and multivariate Cox proportional hazards regression analyses, which yielded consistent results. Nevertheless, residual confounding may persist, and future larger-scale studies should employ PSM or randomized designs to corroborate our findings. Second, as biochemical and immunohistochemical analyses of lipids and lymphocytes were not routinely performed for all patients, several individuals who did not undergo lipid and lymphocyte measurements were excluded. Both blood and tissue levels of cholesterol and NK cells must be assessed to evaluate their influence on CRC prognosis, which may help us to determine whether blood CNR or tissue CNR is more effective in predicting OS. Furthermore, why blood CNR is influenced by gender remains unclear; the regulatory mechanism by cholesterol of NK cells remains largely unknown. Therefore, a large-scale multi-center study and additional experimental analyses are needed to confirm these findings.

## Conclusions

This is the first study to explore the predictive value of CNR, which was an independent risk factor for CRC progression. The nomogram model incorporating CNR and other clinical features may be a valuable tool for predicting survival in patients with CRC.

## Supplementary Material

Supplementary figures and tables.

## Figures and Tables

**Table 1:** Clinicopathological characteristics of 213 patients with CRC.

	Patients (n=213)		
Characteristics	I-II (n=27)	III-IV (n=186)	*P* value
Gender-no. (%)			
Male	21 (77.7%)	120 (64.5%)	0.198
Female	6 (22.3%)	66 (35.5%)	
Age (mean, y)	62.41	61.30	0.661
BMI (mean, kg/m2)	23.31	23.35	0.951
Tumor size (mean, cm)	3.93	4.68	0.029
Tumor location-no. (%)			
Left colon	6 (22.2%)	73 (39.2%)	0.039
Right colon	11 (40.7%)	37 (19.9%)	
Rectum	10 (37.1%)	76 (40.9%)	
Histological type-no. (%)			
Adenocarcinoma	26 (96.3%)	183 (98.4%)	0.421
Others	1 (3.7%)	3 (1.6%)	
Fecal occult blood test-no. (%)			
Positive	25 (92.6%)	168 (90.3%)	0.999
Negative	2 (7.4%)	18 (9.7%)	
Serum CEA-no. (%)			
Positive	14 (51.9%)	136 (73.1%)	0.040
Negative	13 (48.1%)	50 (26.9%)	
Ki67 (mean)	59.63%	67.46%	0.062
Cholesterol (mean, mmol/L)	4.35	4.96	0.017
Triglyceride (mean, mmol/L)	1.12	1.43	0.044
HDL-C (mean, mmol/L)	1.12	1.09	0.729
LDL-C (mean, mmol/L)	2.61	3.09	0.032
PFS (mean, m)	16.33	10.41	0.023

**Table 2:** The correlation between blood CNR and clinicopathological factors.

	Patients (n=213)		
Characteristics	CNR-low (n=110)	CNR-high (n=103)	*P* value
Gender-no. (%)			
Male	81 (73.6%)	60 (58.3%)	0.021
Female	29 (26.4%)	43 (41.7%)	
Age (mean, y)	62.07	60.77	0.437
BMI (mean, kg/m2)	23.62	23.06	0.184
Tumor size (mean, cm)	4.44	4.74	0.199
TNM stage-no. (%)			
I-II	19 (17.3%)	8 (7.8%)	0.041
III-IV	91 (82.7%)	95 (92.2%)	
Tumor location-no. (%)			
Left colon	41 (37.3%)	38 (36.9%)	0.032
Right colon	32 (29.1%)	16 (15.5%)	
Rectum	37 (33.6%)	49 (47.6%)	
Histological type-no. (%)			
Adenocarcinoma	108 (98.2%)	101 (98.1%)	0.999
Others	2 (1.8%)	2 (1.9%)	
Fecal occult blood test-no. (%)			
Positive	102 (92.7%)	91 (88.3%)	0.349
Negative	8 (7.3%)	12 (11.7%)	
Serum CEA-no. (%)			
Positive	82 (74.5%)	68 (66.0%)	0.180
Negative	28 (25.5%)	35 (34.0%)	
Ki67 (mean)	65.09%	67.94%	0.311
PFS (mean, m)	12.42	9.83	0.003

**Table 3:** Univariate and multivariate Cox proportion hazard regression analysis for OS.

Factors	Univariate analysis		Multivariate analysis	
	HR (95% CI)	*P* value	HR (95% CI)	*P* value
Age (≥ 65 vs. < 65)	1.002 (0.978-1.028)	0.858		
Gender (male vs. female)	0.951 (0.486-1.869)	0.883		
Tumor size (≥ 5 cm vs. < 5cm)	1.529 (0.736-3.433)	0.275		
Tumor location (left vs. right)	2.157 (1.083-4.296)	0.021	2.811 (1.414-5.794)	0.004
TNM stage (stage III-IV vs. stage I-II)	2.424 (1.254-4.688)	0.011	2.722 (1.382-5.543)	0.004
tissue NK cells (low vs. high)	1.524 (0.459-4.497)	0.459		
tissue Cholesterol (high vs. low)	1.036 (0.498-2.174)	0.925		
tissue CNR (high vs. low)	2.275 (1.194-4.334)	0.008	2.493 (1.106-5.997)	0.033

**Table 4:** The C-indices of prognostic models for predicting OS.

Models	C-index	95% CI	*P* value
Nomogram	0.737	0.674-0.779	<0.05
Non-tissue CNR nomogram	0.692	0.620-0.765	

## Abbreviations

C-index: concordance index
CEA: carcinoembryonic antigen
CI: confidence interval
CLR: cholesterol-to-lymphocyte ratio
CNR: cholesterol-to-natural killer cell ratio
CRC: colorectal cancer
GEO: gene expression omnibus
GSEA: gene set enrichment analysis
HDL-C: high-density lipoprotein cholesterol
HMGCR: 3-hydroxy-3-methylglutaryl coenzyme A reductase
HR: hazard ratio
LDL-C: low-density lipoprotein cholesterol
LMR: lymphocyte-to-monocyte ratio
MHR: monocyte-to-HDL-C ratio
NK: natural killer
NLR: neutrophil-to-lymphocyte ratio
OS: overall survival
PFS: progression-free survival
PLR: platelet-to-lymphocyte ratio
PPARs: peroxisome proliferator-activated receptors
ROC: receiver operating characteristic
SQLE: squalene monooxygenase
SREBP2: sterol regulatory element- binding protein 2
TCGA: the cancer genome atlas
Th: T helper
TME: tumor microenvironment
TNM: Tumor Node Metastasis
Ts: T suppressor
