# Supplementary material for: The Predictive Value of the Cholesterol-to-Natural Killer Cell Ratio in Colorectal Cancer
Source: J Cancer. 2025 Jun 23;16(9):2946–58. doi: 10.7150/jca.114813 (PMC12244336; doi:10.7150/jca.114813)
Supplement: Supplementary file 1 — Supplementary figures and tables. [file jcav16p2946s1.pdf]

Supplementary Table 1 Clinicopathological characteristics of 94 patients with CRC.

Characteristics	Patients (n=94)
Gender-no. (%)	
Male	48 (51.1%)
Female	46 (48.9%)
Age (mean, y)	64.42
Tumor size (mean, cm)	5.70
Tumor location-no. (%)	
Left colon	42 (44.7%)
Right colon	52 (55.3%)
Histological type-no. (%)	
Adenocarcinoma	93 (98.9%)
Others	1 (1.1%)
Lymphovascular invasion-no. (%)	
Positive	37 (39.4%)
Negative	57 (60.6%)
Perineural invasion-no. (%)	
Positive	17 (18.1%)
Negative	77 (81.9%)
pT category-no. (%)	
T2	7 (7.4%)
T3	35 (37.2%)
T4	52 (55.4%)
pN category-no. (%)	
N0	56 (59.6%)
N1	24 (25.5%)
N2	14 (14.9%)
pM category-no. (%)	
M0	91 (96.8%)
M1	3 (3.2%)
pTNM stage-no. (%)	
Stage I	7 (7.4%)
Stage II	44 (46.8%)
Stage III	40 (42.6%)
Stage IV	3 (3.2%)
tissue Cholesterol-no. (%)	
Low	48 (51.1%)
High	46 (48.9%)
tissue NK cells-no. (%)	

Low	76 (80.9%)
High	18 (19.1%)
Survival-no. (%)	
Alive	57 (60.6%)
Dead	37 (39.4%)

Supplementary Table 2 The correlation between the tissue CNR and clinicopathological factors.

	Patients (n=94)		
Characteristics	CNR-low (n=54)	CNR-high (n=40)	*P* value
Gender-no. (%)			
Male	23 (42.6%)	25 (62.5%)	0.064
Female	31 (57.4%)	15 (37.5%)	
Age (mean, y)	64.43	64.40	0.990
Tumor size-no. (%)			
<5	20 (37.0%)	15 (37.5%)	0.999
≥5	34 (63.0%)	25 (62.5%)	
TNM stage-no. (%)			
I-II	34 (63.0%)	17 (42.5%)	0.061
III-IV	20 (37.0%)	23 (57.5%)	
Tumor location-no. (%)			
Left colon	22 (40.7%)	20 (50.0%)	0.407
Right colon	32 (59.3%)	20 (50.0%)	
Histological type-no. (%)			
Adenocarcinoma	53 (98.1%)	40 (100.0%)	0.999
Others	1 (1.9%)	0 (0.0%)	
Survival-no. (%)			
Alive	40 (74.1%)	17 (42.5%)	0.003
Dead	14 (25.9%)	23 (57.5%)	
OS (mean, m)	74.47	58.7	0.031

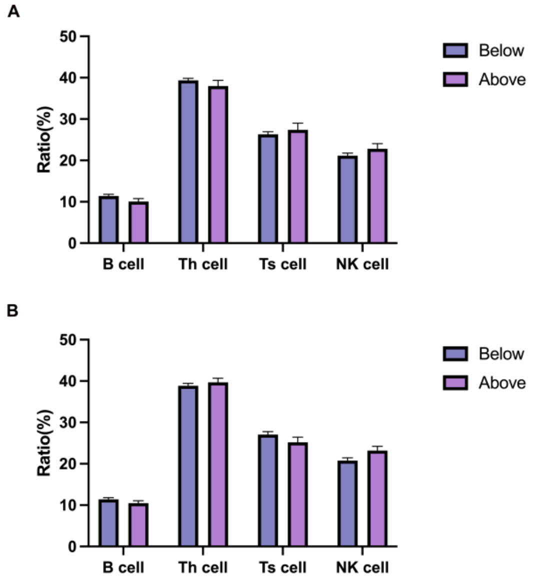

Supplementary Fig. S1. Differences in blood lymphocyte ratios. (A) Differences in lymphocyte ratios between high- and low-triglyceride (blood) patients with CRC. (B) Differences in lymphocyte ratios between high- and low-LDL-C (blood) patients with CRC. Data are expressed as the mean ± SEM. Statistical significance was determined by unpaired Student's t test.

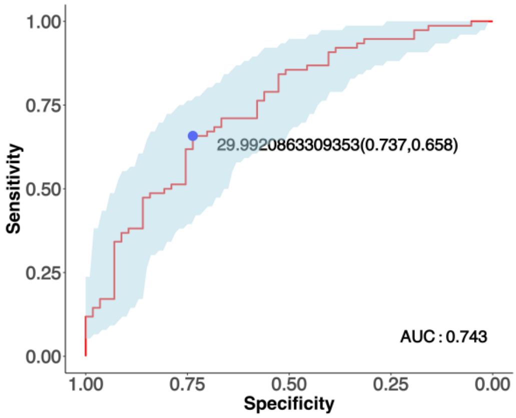

Supplementary Fig. S2. The optimal blood CNR cut-off point.

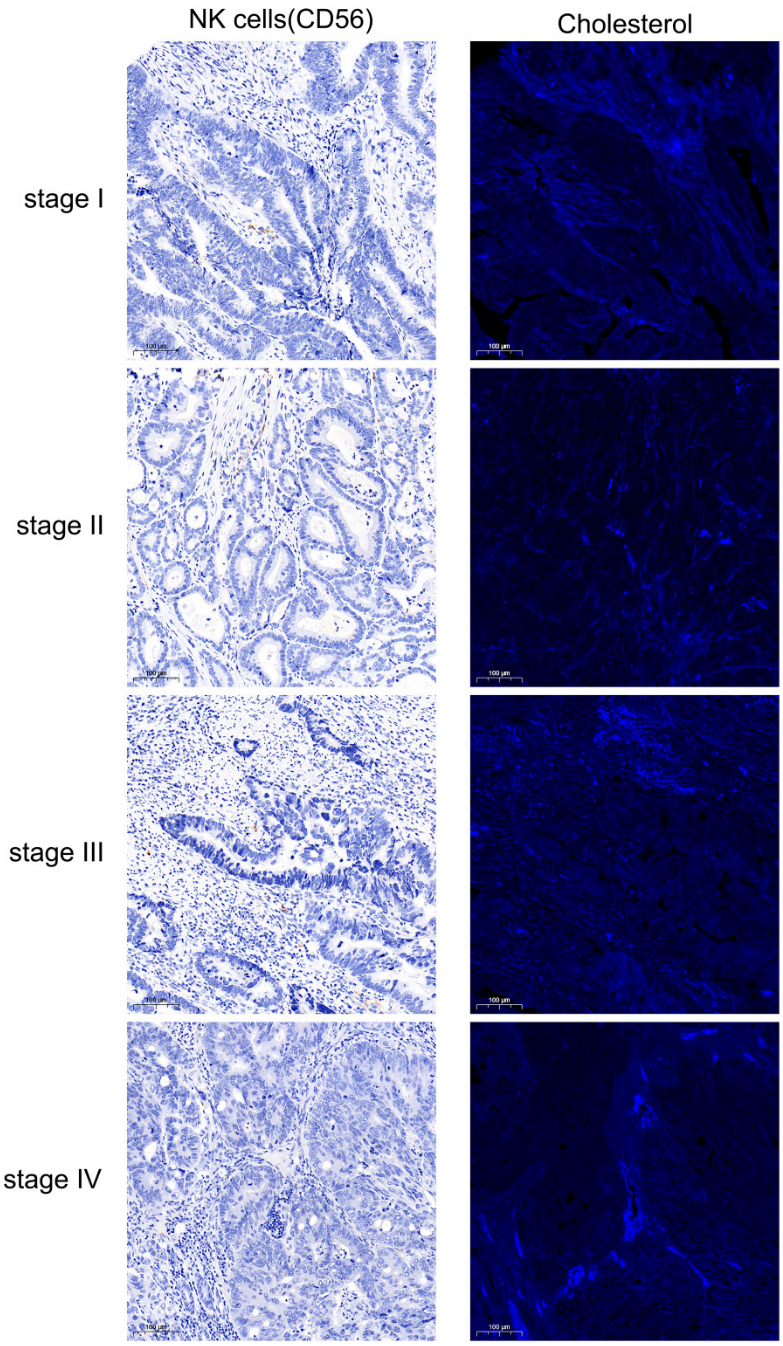

Supplementary Fig. S3. The NK cells (tissue, immunohistochemical staining) and cholesterol (tissue, immunofluorescence staining) expression profiles of different TNM stages (100× magnifications).

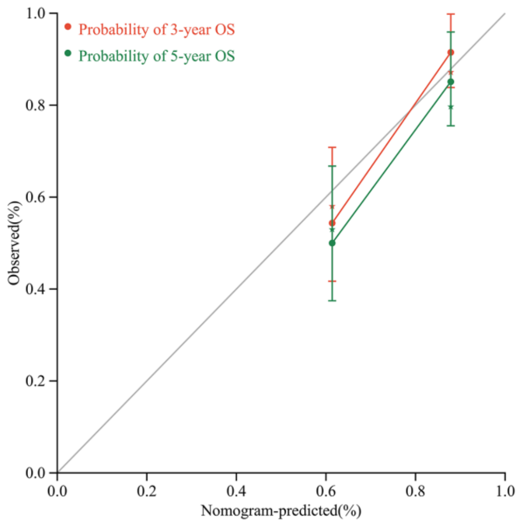

Supplementary Fig. S4. Calibration curves of the prognostic nomogram for predicting 3-year (red) and 5-year (green) OS in patients with CRC. The Y-axis represents the actual OS; the X-axis represents the nomogram-predicted OS.

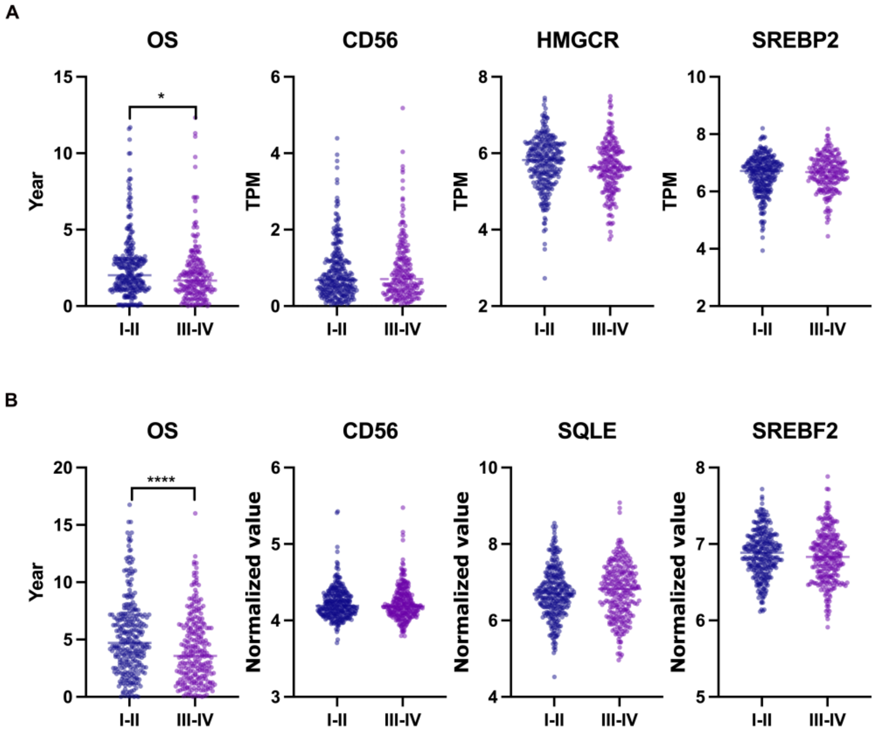

Supplementary Fig. S5. Gene expression of cholesterol synthesis and NK cells in colon cancer datasets. (A) Compared to TNM stage I-II, *CD56*, *HMGCR*, and *SREBP2* gene expression was unchanged in the TNM stage III-IV group (TCGA). (B) Compared to TNM stage I-II, *CD56*, *SQLE*, and *SREBP2* gene expression was unchanged in the TNM stage III-IV group (GSE39582). Statistical significance was determined by unpaired Student's t test. *, $P < .05$; ****, $P < .0001$.
